# Clinical Utility of Lipoprotein(a) and *LPA* Genetic Risk Score in Risk Prediction of Incident Atherosclerotic Cardiovascular Disease

**DOI:** 10.1001/jamacardio.2020.5398

**Published:** 2020-10-06

**Authors:** Mark Trinder, Md Mesbah Uddin, Phoebe Finneran, Krishna G. Aragam, Pradeep Natarajan

**Affiliations:** 1 Centre for Heart Lung Innovation, The University of British Columbia, Vancouver, British Columbia, Canada; 2 Program in Medical and Population Genetics and the Cardiovascular Disease Initiative, Broad Institute of Harvard, Cambridge, Massachusetts; 3 Massachusetts General Hospital, Cardiology Division, Harvard Medical School, Boston, Massachusetts; 4 Cardiovascular Research Center, Massachusetts General Hospital, Boston; 5 Massachusetts General Hospital, Department of Medicine, Harvard Medical School, Boston

## Abstract

**Importance:**

Lipoprotein(a) is a highly heritable biomarker independently associated with atherosclerotic cardiovascular disease (ASCVD). It is unclear whether measured lipoprotein(a) or genetic factors associated with lipoprotein(a) can provide comparable or additional prognostic information for primary prevention.

**Objective:**

To determine whether a genetic risk score (GRS) comprising 43 variants at the *LPA* gene, which encodes apolipoprotein(a), has clinical utility in assessing ASCVD risk compared with and in addition to lipoprotein(a) measurement.

**Design, Setting, and Participants:**

The UK Biobank is a prospective observational study of approximately 500 000 volunteers aged 40 to 69 years who were recruited from 22 sites across the United Kingdom between 2006 and 2010. Using externally derived weights, an *LPA* GRS was calculated for 374 099 unrelated individuals with array-derived genotypes and lipoprotein(a) measures. Data were analyzed from April 2020 to March 2020.

**Exposures:**

Measured lipoprotein(a) and *LPA* GRS.

**Main Outcomes and Measures:**

We estimated the associations between measured lipoprotein(a) and *LPA* GRS with the incidence of ASCVD (peripheral arterial disease, coronary artery disease, myocardial infarction, ischemic stroke, and cardiovascular mortality) using Cox proportional hazards models. To determine the utility of using measured lipoprotein(a) and *LPA* GRS as risk enhancers for ASCVD, we assessed the potential improvement in ASCVD risk discrimination by QRISK3 and Pooled Cohort Equations among individuals with borderline to intermediate risk (n = 113 703 and 144 350, respectively).

**Results:**

The mean age of the overall study population was 57.6 years, and 204 355 individuals were female (54.6%). During a median follow-up of 11.1 years (interquartile range, 1.4 years), 15 444 individuals developed an incident ASCVD event (5.1%). The *LPA* GRS explained approximately 60% of the variation in measured lipoprotein(a) for White/European individuals. Independently, both lipoprotein(a) and *LPA* GRS were associated with incident, composite ASCVD (hazard ratio per 120 nmol/L increase, 1.26; 95% CI, 1.23-1.28 vs hazard ratio, 1.29; 95% CI, 1.26-1.33; *P* < .001). The association between *LPA* GRS and ASCVD was substantially attenuated after adjusting for measured lipoprotein(a). Adding measured lipoprotein(a) or *LPA* GRS to QRISK3 provided modest improvements to the risk discrimination of incident ASCVD events (area under the receiver operating curve, 0.640; 95% CI, 0.633-0.647 vs 0.642; 95% CI, 0.635-0.649 for both; *P* = .005 and *P* = .01, respectively).

**Conclusions and Relevance:**

When indicated, cardiovascular risk assessment with lipoprotein(a) at middle-age may include direct measurement or an *LPA* GRS.

## Key Points

**Question:**

Does measurement of lipoprotein(a) and/or *LPA* genetic risk score (GRS) have clinical utility in risk prediction of incident atherosclerotic cardiovascular disease (ASCVD)?

**Findings:**

In this cohort of 283 540 adults recruited by the UK Biobank, both measured lipoprotein(a) and *LPA* GRS were associated with comparable risk of incident ASCVD events. The *LPA* GRS did not yield additional prognostic information beyond measured lipoprotein(a), and both measured lipoprotein(a) and *LPA* GRS yielded modest improvements in the discrimination of ASCVD risk relative to the Pooled Cohort Equations or QRISK3.

**Meaning:**

Cardiovascular risk assessment with lipoprotein(a) may be achieved with either direct measurement or an *LPA* GRS.

## Teaser

This study investigates whether a genetic risk score comprising 43 variants at the *LPA* gene, which encodes apolipoprotein(a), has clinical utility in assessing atherosclerotic cardiovascular disease risk compared with and in addition to lipoprotein(a) measurement.

## Introduction

Lipoprotein(a) is a plasma lipoprotein composed of a low-density lipoprotein (LDL) particle that is covalently linked to apolipoprotein(a) by a disulfide bond. On an equimolar basis, lipoprotein(a) is more atherogenic than LDL because the additional apolipoprotein(a) component may exacerbate atherothrombosis by promoting vascular inflammation and its potential antifibrinolytic activity is associated with inhibition of plasminogen.^1,2,3^ Lipoprotein(a) levels are 75% to 95% heritable and predominately determined by single-nucleotide variants at the *LPA* gene and copy number variants specifically in the kringle IV type 2 domain.^4,5,6^ Genetic association studies have identified genetic variants explaining approximately 60% of the variability in lipoprotein(a) levels in European populations.^7,8^ Elevated lipoprotein(a), defined as lipoprotein(a) level of 120 nmol/L or greater or approximately 50 mg/dL, is common and varies in prevalence by ancestry (affects 1 in 5 European individuals).^9,10,11,12^

Mendelian randomization studies^7,13^ suggest that lipoprotein(a) is a causal contributor to ASCVD,^7,13^ and ongoing randomized clinical trials are testing this hypothesis.^14,15,16^ Both the diagnostic yield and clinical value of genetic testing of *LPA* are not well understood. Specifically, unlike LDL cholesterol, it remains unclear whether genetic factors related to lipoprotein(a) may provide information regarding lifetime exposure relevant to ASCVD risk prediction and selection of patients for primary prevention with statin therapy.^4,17,18,19^

Here, we report our investigation of the prognostic utility of testing for the genetic determinants of lipoprotein(a) relative to direct measurement of lipoprotein(a) levels in a large cohort of middle- to late-age adults not using lipid-lowering medication and without baseline ASCVD.

## Methods

### UK Biobank Cohort

**Figure 1.**
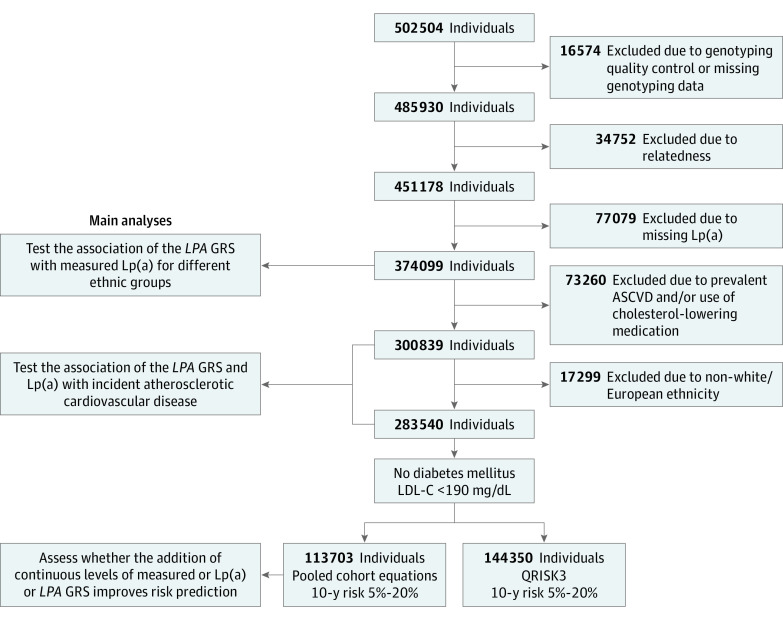
Flow Diagram of Analyses ASCVD indicates atherosclerotic cardiovascular disease; GRS, genetic risk score; Lp(a), lipoprotein(a); LDL-C, low-density lipoprotein cholesterol. To convert LDL-C to millimoles per liter, multiply by 0.0259; Lp(a) to milligrams per deciliter, divide by 2.4.

The UK Biobank is a prospective observational study of approximately 500 000 volunteer adults aged 40 to 69 years recruited from 22 sites across the United Kingdom between 2006 and 2010, with follow-up ongoing. This study included individuals of third-degree relatedness or less (Figure 1).^20^ Biochemical measurements, physical examination measurements, and medical histories were assessed at the time of study enrollment. Self-reported ethnicities were categorized as mixed, African/Black, European/White, East Asian, South Asian, and unknown.

The UK Biobank protocol was approved by the Northwest Multi-Center Research Ethics Committee, and all study participants provided written informed consent. Secondary use of data for this study was approved by the Massachusetts General Hospital institutional review board 2013P001840.

### Lipoprotein(a) Measurement

Lipoprotein(a) was measured in nanomoles per liter at study enrollment using an immunoturbidimetric method on the Beckman Coulter AU5800 platform (Randox Bioscience, UK), which is isoform insensitive.^10^ To convert lipoprotein(a) values to milligrams per deciliter, divide by 2.15.

### *LPA* Genetic Risk Score

We used genotyping array data from UK Biobank participants to calculate a previously described weighted *LPA* genetic risk score (GRS), composed of 43 single-nucleotide variants that were conditionally and significantly associated with lipoprotein(a) levels in data sets external to the UK Biobank (eTable 1 and eMethods in the Supplement).^7^ The units of the *LPA* GRS were converted from milligrams per deciliter to nanomoles per liter by multiplying by 2.15 to harmonize units with the measured lipoprotein(a).^21^ An *LPA* GRS of at least 120 was considered elevated.^7,8,11,12,17,22^

### Atherosclerotic Cardiovascular Disease Outcomes

The outcomes of myocardial infarction, coronary artery disease, ischemic stroke, peripheral arterial disease, cardiovascular mortality, and a composite of the aforementioned outcomes were based on International Statistical Classification of Diseases and Related Health Problems and Office of Population Censuses and Surveys Classification of Interventions and Procedures codes (eTable 2 in the Supplement).^23^ Incident events were defined as the first event occurring between the date of enrollment and the end of follow-up of March 31, 2020. Individuals were censored at death or loss to follow-up. Individuals reporting use of cholesterol-lowering medication or prevalent angina, coronary artery disease, history of myocardial infarction, history of stroke, or peripheral arterial disease/revascularization at study enrollment were excluded from analyses of incident events. A sensitivity analysis was performed with individuals without prevalent ASCVD but using cholesterol-lowering medication.

### Statistical Analyses

Analyses were performed using R, version 4.0.0 (R Core Team [2020]). The association between *LPA* GRS and measured lipoprotein(a) levels were assessed by Spearman correlation and adjusted linear regression models (covariates of age, sex, assessment center, genotyping batch, and the first 5 principal components of ancestry).

We assessed the risk of incident ASCVD events Cox proportional hazards models with the covariates of sex, age, assessment center, genotyping batch, and the first 5 principal components of ancestry using the survival package (version 3.1-12).^24^ When explicitly stated, Cox proportional hazards models included the additional covariate of continuous, measured lipoprotein(a) or *LPA* GRS.

To compare whether an elevated *LPA* GRS was associated with greater risk of ASCVD for a given lipoprotein(a) level, individuals with and without an elevated *LPA* GRS were matched at a 1:1 ratio based on measured lipoprotein(a) levels using the nearest neighbor algorithm from the MatchIt, version 3.0.2 (R Core Team [2020]), package with a caliper set to 0.02.^25^ Measured lipoprotein(a) levels were compared between matched individuals with and without an elevated *LPA* GRS using a Mann-Whitney *U* test. The risks of incident ASCVD events were compared between matched individuals with and without an elevated *LPA* GRS using a log-rank test.

We calculated the incidence rates for composite ASCVD events for each percentile of measured lipoprotein(a) and *LPA* GRS using the Poisson rate exact method from the epitools package, version 0.5-10.1.^26^ Additionally, for individuals with and without an elevated *LPA* GRS (≥120 nmol/L), we calculated the incidence rates for composite ASCVD events for percentiles of measured lipoprotein(a) and *LPA* GRS or for 50 nmol/L bins of measured lipoprotein(a) levels with the maximum lipoprotein(a) bin was set to lipoprotein(a) levels 250 nmol/L or greater.

Lastly, we assessed whether lipoprotein(a) levels and/or *LPA* GRS could refine the ASCVD risk discrimination for individuals without diabetes or severe hypercholesterolemia (LDL cholesterol level ≥190 mg/dL; to convert to millimoles per liter, multiply by 0.0259) classified as borderline-intermediate risk by the Pooled Cohort Equations^27,28^ and QRISK3,^29^ respectively (predicted 10-year ASCVD risk: 5%-20%). This borderline-intermediate risk group consists of individuals in whom statin therapy could be warranted if risk enhancers are present for a given patient (ie, elevated lipoprotein[a]).^27,28^ We used multiple imputation by chained equations to impute missing values needed to calculate ASCVD risk according to the Pooled Cohort Equations and QRISK3 with the mice package, version 3.11.0 (The R Foundation).^30^ The associations between continuous, measured lipoprotein(a) levels, *LPA* GRS, and Pooled Cohort Equations–derived or QRISK3-derived clinical risk scores^27^ with incident myocardial infarction, ischemic stroke, or cardiovascular mortality were assessed by the area under the curve from receiver operating characteristic models^31^ and the Harrell C statistic^32^ from Cox proportional hazards models. We assessed whether Cox proportional hazards models and correlated receiver operating characteristic curves were statistically different using an analysis of variance test and Delong test, respectively. Statistical significance was claimed when 2-sided *P* values were less than .05.

## Results

### Cohort Characteristics

A flowchart of the subgroups used in this study is shown in Figure 1. For the overall study population of 374 099 individuals, the mean (SD) age at study enrollment was 57.6 (8.0) years, and 204 355 individuals were female (54.6%; Table 1). The distributions of measured lipoprotein(a), *LPA* GRS, and the residual of measured vs *LPA* GRS-expected lipoprotein(a) for these individuals are shown across strata of ethnicity in eFigures 1 and 2 in the Supplement. The *LPA* GRS was associated with measured levels of lipoprotein(a) for White/European individuals (*R^2^* = 0.595; *P* < .001), South Asians (*R^2^* = 0.11; *P* < .001), East Asian individuals (*R^2^* = 0.078; *P* < .001), and Black/African individuals (*R^2^* = 0.038; *P* = .03). The Pearson correlation coefficient between the *LPA* GRS and measured lipoprotein(a) levels was 0.717 for White/European individuals, 0.371 for South Asian individuals, 0.281 for East Asian individuals, and 0.070 for Black/African individuals (eTable 3 in the Supplement).

**Table 1.** Enrollment Characteristics of UK Biobank Study Population

Characteristic	No. (%)	
	White/European and non–White/European	European
No.	374 099	350 903
Age, mean (SD), y	57.6 (8.0)	57.9 (8.0)
Female sex	204 355 (54.6)	191 967 (54.7)
Ethnicity		
Mixed	2340 (0.6)	NA
African/Black	6521 (1.7)	NA
East Asian	2774 (0.7)	NA
European	350 903 (93.8)	350 903 (100.0)
South Asian	6203 (1.7)	NA
Unknown	5358 (1.4)	NA
Total cholesterol, mean (SD) [No.], mg/dL	221.1 (44.4) [373 827]	221.9 (44.3) [350 647]
Direct LDL-C, mean (SD) [No.], mg/dL	138.3 (33.7) [373 228]	138.8 (33.7) [350 091]
Triglycerides, median (IQR) [No.], mg/dL	130.8 (96.8) [373 807]	131.4 (96.6) [350 632]
HDL-C, mean (SD) [No.], mg/dL	56.1 (14.8) [342 514]	56.3 (14.8) [321 239]
Lipoprotein(a), median (IQR) [No.], nmol/L	25.1 (79.3)	24.0 (78.8)
Lipoprotein(a) ≥120 nmol/L	71 957 (19.2)	67 676 (19.3)
C-reactive protein, median (IQR) [No.], g/L	1.3 (2.1) [373 035]	1.3 (2.1) [349 912]
Hemoglobin A_1c_, median (IQR) [No.], % of total hemoglobin	35.2 (5.1) [355 971]	35.2 (5.0) [335 115]
Cholesterol-lowering medication, No./total No. (%)	65 357/370 833 (17.6)	60 948/348 768 (17.5)
Antihypertensive medication, No./total No. (%)	77 923/370 833 (21.0)	72 217/348 768 (20.7)
Angina	12 072 (3.2)	11 275 (3.2)
Coronary revascularization	6562 (1.8)	6163 (1.8)
Myocardial infarction	8661 (2.3)	8144 (2.3)
Ischemic stroke	5653 (1.5)	5345 (1.5)
Peripheral arterial disease	1011 (0.3)	975 (0.3)
Peripheral arterial revascularization	605 (0.2)	573 (0.2)
Hypertension, No./total No. (%)	100 848/372 730 (27.1)	94 102/350 302 (26.9)
Diabetes, No./total No. (%)	18 916/372 461 (5.1)	16 195/350 098 (4.6)
BMI, mean (SD) [No.]	27.4 (4.8) [372 578]	27.4 (4.7) [349 772]
Current smoker, No./total No. (%)	38 998/372 169 (10.5)	36 283/349 645 (10.4)

Abbreviations: BMI, body mass index (calculated as weight in kilograms divided by height in meters squared); HDL-C, high-density lipoprotein cholesterol; IQR, interquartile range; LDL-C, low-density lipoprotein cholesterol.

SI conversion factors: To convert cholesterol levels to millimoles per liter, multiply by 0.0259; C-reactive protein to milligrams per liter, multiply by 10; hemoglobin A_1c_ to proportion of total hemoglobin, multiply by 0.01; lipoprotein(a) to milligrams per deciliter, divide by 2.4.

For 300 839 individuals without prevalent ASCVD and not using cholesterol-lowering medication, the mean (SD) age at study enrollment was 56.6 (8.0) years, and 174 555 individuals were female (58.0%). The median level of lipoprotein(a) was 24.1 nmol/L (interquartile range, 73.6 nmol/L) and the median length of follow-up for composite ASCVD events was 11.1 years (interquartile range, 1.4 years; eTable 4 in the Supplement). A 120-nmol/L increase in measured lipoprotein(a) was significantly associated with increased risk of incident, composite ASCVD events for White/European individuals (hazard ratio [HR], 1.25; 95% CI, 1.23-1.27; *P* = 3.71 × 10^−71^), South Asian individuals (HR, 1.24; 95% CI, 1.03-1.44; *P* = .04), East Asian individuals (HR, 1.76; 95% CI, 1.27-2.25; *P* = .02), and mixed individuals (HR, 1.39; 95% CI, 1.06-1.71; *P* = .05), but not Black/African individuals (HR, 1.06; 95% CI, 0.84-1.30; *P* = .58) or individuals of unknown ethnicity (HR, 1.11; 95% CI, 0.84-1.40 *P* = .44; eFigure 3 in the Supplement). For statistical power and predictive strength, we focused subsequent analyses on individuals of White/European ethnicity (Figure 1).

### Association of Measured Lipoprotein(a) and *LPA* GRS With Atherosclerotic Cardiovascular Diseases

**Figure 2.**
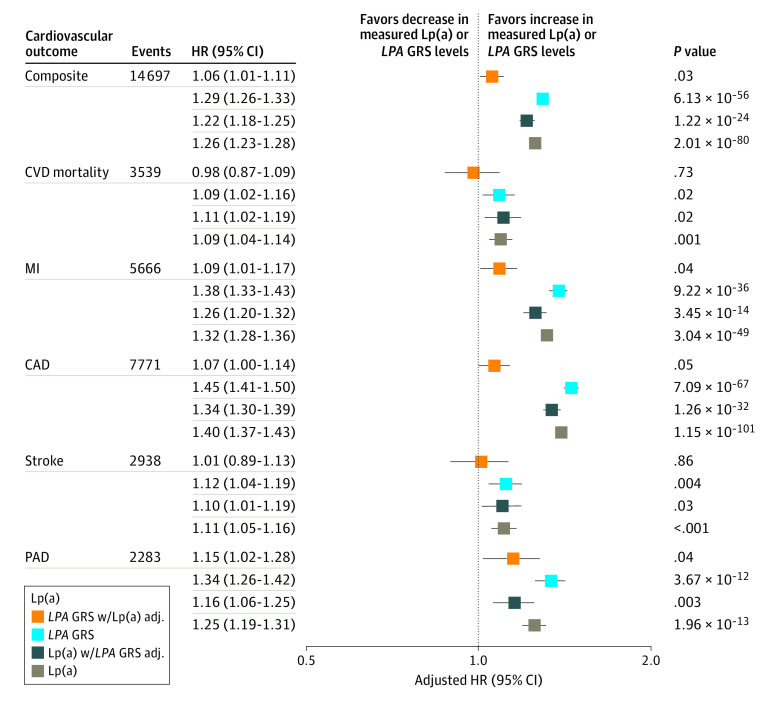
Association of Measured Lipoprotein(a) (Lp[a]) and *LPA* Genetic Risk Score (GRS) With Incident Atherosclerotic Cardiovascular Disease (ASCVD) Among Individuals of White/European Ethnicity The risk of incident ASCVD events is depicted as hazard ratios (HRs) and 95% confidence intervals for peripheral arterial disease (PAD), ischemic stroke, coronary artery disease (CAD), myocardial infarction (MI), cardiovascular disease (CVD) mortality, and composite ASCVD for 292 963 individuals not using cholesterol-lowering medication and without prevalent ASCVD at enrollment. Hazard ratios are scaled to depict a 120-nmol/L increase in measured Lp(a) or *LPA* genetic risk score (GRS) levels. All Cox proportional hazard models included age, sex, assessment center, genotyping batch, and the first 5 principal components of ancestry. Adj indicates adjusted.

For individuals of White/European ethnicity without prevalent ASCVD and not using cholesterol-lowering medication (n = 283 540), a 120-nmol/L increase in either measured lipoprotein(a) or *LPA* GRS was associated with increased risk of incident peripheral arterial disease (HR, 1.25; 95% CI, 1.19-1.31 and 1.34; 95% CI, 1.26-1.42), coronary artery disease (HR, 1.40; 95% CI, 1.37-1.43 and 1.45; 95% CI, 1.41-1.50), myocardial infarction (HR, 1.32; 95% CI, 1.28-1.36 vs 1.38, 95% CI, 1.33-1.43), ischemic stroke (HR, 1.11; 95% CI, 1.05-1.16 and 1.12, 95% CI, 1.04-1.19), and cardiovascular mortality (HR, 1.09; 95% CI, 1.04-1.16 and 1.09, 95% CI, 1.02-1.16; Figure 2). In a sensitivity analysis of individuals of White/European ethnicity without prevalent ASCVD but using cholesterol-lowering medication (n = 43 829), the effect estimates for the association between lipoprotein(a) and risk of incident ASCVD were modestly attenuated (eFigure 4 in the Supplement). Notably, the risk of incident, composite ASCVD events and probability of ASCVD by age 75 years was more pronounced at the extremes of measured lipoprotein(a) than *LPA* GRS (eFigure 5 in the Supplement).

### Using *LPA* GRS for Cardiovascular Disease Risk Prediction Compared With Measured Lipoprotein(a) Levels

**Figure 3.**
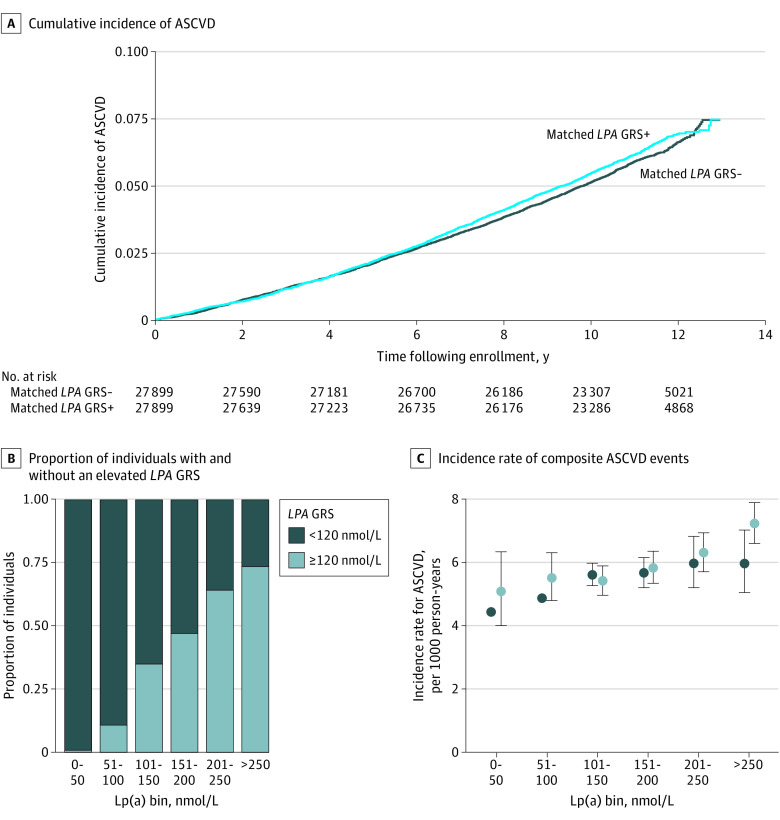
Association of Elevated *LPA* Genetic Risk Score (GRS) With Cardiovascular Risk When Individuals Are Matched for Measured Lipoprotein(a) (Lp[a]) Levels A, Crude time-to-first incident, composite atherosclerotic cardiovascular disease (ASCVD) event curves are shown for individuals with and without an elevated *LPA* GRS after matching for Lp(a) levels (matched *LPA* GRS-: predicted Lp(a) <120 nmol/L; matched *LPA* GRS+: predicted Lp(a) ≥120 nmol/L). B, The proportion of individuals with and without an elevated *LPA* GRS. C, Incidence rate of composite ASCVD events per 1000 person-years are displayed for 50-nmol/L bins of measured Lp(a) levels. Data points for incidence rates are displayed with the 95% confidence interval stratified by elevated *LPA* GRS cutoff levels of 120 nmol/L.

The associations between *LPA* GRS and risk of incident peripheral arterial disease, coronary artery disease, myocardial infarction, ischemic stroke, cardiovascular mortality, and composite ASCVD were substantially attenuated when models were additionally adjusted for measured lipoprotein(a) levels (Figure 2). Additionally, to assess the association of an elevated *LPA* GRS with ASCVD risk at comparable levels of measured lipoprotein(a), we matched individuals of White/European ethnicity with and without an elevated *LPA* GRS (≥120 nmol/L) based on measured lipoprotein(a). Measured lipoprotein(a) levels were comparable between matched individuals with and without elevated *LPA* GRS (median, 149.2 nmol/L; interquartile range, 74.3 nmol/L vs median, 149.1 nmol/L; interquartile range, 74.0 nmol/L; Mann-Whitney *P* = .56). There was no significant difference in the risk of an incident, composite ASCVD events between individuals with and without an elevated *LPA* GRS matched for lipoprotein(a) levels (log-rank *P* = .60; Figure 3A).

To further explore the influence of an elevated *LPA* GRS on risk of ASCVD at comparable levels of lipoprotein(a), we assessed the incidence rate of composite ASCVD events across 50-nmol/L bins of measured lipoprotein(a) for individuals of White/European ethnicity with and without an elevated *LPA* GRS (≥120 nmol/L). In general, there was a trend toward increased incidence rate of ASCVD with increasing bins of lipoprotein(a) levels for both individuals with and without an elevated *LPA* GRS. However, for each elevated *LPA* GRS threshold, an elevated lipoprotein(a) GRS did not significantly associate with a greater incidence rate of ASCVD compared with a nonelevated *LPA* GRS within a given bin of measured lipoprotein(a) (Figure 3B and C).

### Lipoprotein(a) and Improvement in Risk Discrimination Among Borderline-Intermediate Risk Patients

The distribution of QRISK3 and Pooled Cohort Equations 10-year ASCVD risk scores for the 300 839 individuals of European and non-European ethnicity not using cholesterol-lowering medication and without prevalent ASCVD at enrollment are displayed in eFigure 6 in the Supplement. Prediction with QRISK3 had significantly greater ASCVD risk discrimination compared with the Pooled Cohort Equations for these individuals (eTable 5 in the Supplement). We used a subgroup of 113 703 and 144 350 individuals of White/European ethnicity defined as borderline to intermediate ASCVD risk by the Pooled Cohort Equation and QRISK3 to assess the role of measured lipoprotein(a) and *LPA* GRS as a risk enchancer.^12,27^ Adding continuous, measured lipoprotein(a) levels to QRISK3 provided modest improvements to the predicted risk of incident ASCVD events as assessed by AUROC and Harrell C statistic (Table 2). The AUROC improved from 0.640 (95% CI, 0.633-0.647) to 0.642 (95% CI, 0.634-0.649) and 0.642 (95% CI, 0.634-0.649) when measured, continuous lipoprotein(a) levels and *LPA* GRS were added to QRISK3 (*P* = .005 and *P* = .01, respectively). Similar results were observed when continuous, measured lipoprotein(a) was added to the Pooled Cohort Equations and when analyses were restricted to only individuals with complete clinical data (no imputation and exclusion of individuals with missing data; eTables 6-8 in the Supplement).

**Table 2.** Additional Value of Lp(a) Metrics to Atherosclerotic Cardiovascular Disease Risk Prediction Among Individuals of European Ethnicity Defined by QRISK3 Using Complete and Imputed Data^a^

Features	AUROC (95% CI)	*P* value	Harrell C statistic (SE)	*P* value
QRISK3	0.640 (0.633-0.647)	1 [Reference]	0.639 (0.004)	1 [Reference]
QRISK3 and Lp(a)	0.642 (0.635-0.649)	.005	0.641 (0.004)	5.26 × 10^−10^
QRISK3 and *LPA* GRS	0.642 (0.634-0.649)	.01	0.641 (0.004)	2.26 × 10^−9^
QRISK3 and Lp(a) and *LPA* GRS	0.642 (0.635-0.649)	.005	0.641 (0.004)	7.49 × 10^−10^

Abbreviations: ASCVD, atherosclerotic cardiovascular disease; AUROC, area under the receiver operating characteristic curve; GRS, genetic risk score; PCE, pooled cohort equations.

^a^ This subgroup included 144 350 individuals classified as having borderline-intermediate ASCVD risk without prevalent ASCVD, diabetes mellitus, severe hypercholesterolemia, or use of cholesterol-lowering medication (10-year risk of 5%-20%). A total of 5505 individuals experienced a myocardial infarction, an ischemic stroke, or cardiovascular mortality event over a median follow-up of 11.1 years (interquartile range 1.4 years). The AUROC and the Harrell C statistic for Cox proportional hazards models are shown for the PCE with and without the addition of continuous measured lipoprotein(a) and *LPA* GRS. Models were compared relative to the QRISK3 model using an analysis of variance test for Cox proportional hazard models or DeLong test for receiver operating characteristic curves.

## Discussion

Here we used the UK Biobank cohort to demonstrate that measured lipoprotein(a) and an *LPA* GRS are associated with increased risk of incident ASCVD in a primary prevention setting. The major advance from this study is the demonstration that an *LPA* GRS offered comparable ASCVD risk prediction to directly measured lipoprotein(a). In a large observational cohort, we observed that increased lipoprotein(a) is independently associated with future risk for ASCVD but with modest discrimination in addition to clinical risk scores at the level of population screening for primary prevention.

In contrast to LDL cholesterol, this work suggests that profiling the genetic determinants of plasma lipoprotein(a) does not provide additional value to measured levels of lipoprotein(a) in terms of ASCVD risk prediction. These data suggest that the *LPA* GRS is likely not a better marker for exposure to lipoprotein(a) than an isolated lipoprotein(a) measurement. We suspect that this is explained by 2 key factors: (1) lipoprotein(a) displays higher heritability than LDL cholesterol levels, and (2) lipoprotein(a) levels are generally much more stable throughout life compared with other circulating lipoproteins (ie, minimal influence of age, sex, genetic factors outside the *LPA* gene, environmental factors, or currently available medicines).^33^ The polygenic architecture of complex traits, such as lipoprotein(a), have shown variable transferability across genetic ancestries as demonstrated in this study and others.^4,18,34,35,36,37^ Elevated lipoprotein(a) is strongly associated with ASCVD risk among multiple ethnic groups, and lipoprotein(a) levels greater than 120 nmol/L are observed in 1 of 4 African individuals, 1 in 10 South and South East Asian individuals, 1 in 10 Arab individuals, 1 in 7 Latin American individuals, and 1 in 5 European individuals.^35,38,39,40^ We observed that risk prediction performance for *LPA* GRS and measured lipoprotein(a) varies similarly across ethnicities.

Our findings are in accordance with other studies demonstrating that adding continuous lipoprotein(a) levels to clinical risk scores leads to modest improvements in risk discrimination for ASCVD.^22,41,42^ While some guidelines support broad population-based screening with lipoprotein(a),^43^ the small improvement in the C statistics relative to clinical risk scores (Δ C statistic of approximately 0.002) suggests that measured lipoprotein(a) or *LPA* GRS may be an inefficient approach for refinement of ASCVD risk among asymptomatic middle-aged adults broadly.^44^ However, because lipoprotein(a) levels display an extremely right-skewed distribution in the general population (potentially varying more than 1000-fold between individuals, approximately 0.2 to ≥200 mg/dL), individuals with extreme lipoprotein(a) levels greater than 200 mg/dL could have a 3- to 4-fold increased lifetime risk of ASCVD.^7,13,17,45^ In such cases, the modest improvement in ASCVD risk discrimination that was observed in this study when continuous levels of measured lipoprotein(a) or *LPA* GRS were added to clinical risk scores may underestimate cardiovascular risk. Inclusion of the entire range of lipoprotein(a) levels in ASCVD risk discrimination and reclassification models often fails to accurately quantify the cardiovascular risk associated with the extremes of elevated lipoprotein(a) (ie, because extremely elevated lipoprotein[a] is rare there is limited predictive power).^17,41^ Efficient strategies to identify individuals with extremely elevated lipoprotein(a) levels require further research.

Previous studies suggest that small apolipoprotein(a) isoforms, which are also associated with higher lipoprotein(a) levels, may increase an individual’s risk of ASCVD more than expected by changes in lipoprotein(a) concentration alone.^46^ However, in this study we did not find that the *LPA* GRS, which contains several genetic variants associated with small apolipoprotein(a) size,^6^ explained additional ASCVD risk beyond measured levels of lipoprotein(a). Unlike isoform-independent lipoprotein(a) molar concentration, the associations of apolipoprotein(a), and by extension isoform-dependent measurements, with ASCVD may be confounded by pleiotropic effects on high-density lipoprotein cholesterol and triglycerides.^19^ In some cases, the genetic determinants of elevated lipoprotein(a) may help discern the familial risk of lipoprotein(a)-associated ASCVD that is not always conclusive from lipoprotein(a) measurement alone. For instance, a single copy of the rs10455872-G allele, which is common in European populations, is known to associate with extremely elevated lipoprotein(a) that can present phenotypically similar to familial hypercholesteremia and thus may be amenable to cascade screening.^47,48^

### Limitations

First, a major limitation of this study is that it focused on individuals of White/European ethnicity, and the generalizability of these findings, in particular the *LPA* GRS, to other ethnic groups requires further research. Notably, we show that the association of measured lipoprotein(a) with incident ASCVD risk may vary between ethnic groups and that, similar to other GRSs, the *LPA* GRS performed suboptimally in non–White/European ethnicities. An *LPA* GRS needs to be developed and tested in non–White/European cohorts to determine whether these findings extend to individuals of other ethnic groups. Second, most individuals enrolled in this study were recruited at middle age (mean [SD], 56.8 [8.0] years), with median follow-up of 11.1 years, and thus the influence of measured lipoprotein(a) and *LPA* GRS on incident premature and lifelong ASCVD risk remains to be determined in prospective primary prevention cohorts with younger age of enrollment and longer follow-up. Third, additional limitations include the ascertainment bias associated with volunteer recruitment, use of hospitalization and operation codes to define ASCVD events, and that the *LPA* GRS was constructed with winsorization (lipoprotein[a] levels greater than 130 mg/dL were set to 130 mg/dL), which may fail to explain some of the genetic variation associated with extremely elevated levels of measured lipoprotein(a).^7^ Despite this, the *LPA* GRS is still a reasonable genetic predictor that explains greater than 60% of the variation in lipoprotein(a) levels, an association that is considerably greater than contemporary GRSs for other lipid traits such as LDL cholesterol.^49^

## Conclusions

In conclusion, the results from this study suggest that an *LPA* GRS provides comparable risk prediction for incident ASCVD compared with measured lipoprotein(a). Measured lipoprotein(a) and the *LPA* GRS both provided modest improvement in risk discrimination beyond guideline-supported risk scores, which supports the role of lipoprotein(a) as a risk-enhancing factor.

## Notes

Supplement. **eMethods.** **eTable 1. **Genetic variants used in the LPA genetic risk score. **eTable 2. **International Statistical Classification of Diseases and Related Health Problems (ICD) and Office of Population Censuses and Surveys Classification of Interventions and Procedures (OCPS) codes used to define atherosclerotic cardiovascular disease events. **eTable 3. **Spearman's rank correlation between LPA genetic risk score and measured lipoprotein(a) levels for different ethnic groups. **eTable 4. **Enrollment characteristics of UK Biobank study participants not using cholesterol-lowering medication and without prevalent atherosclerotic cardiovascular disease at study enrollment. **eTable 5. **Risk discrimination models for incident atherosclerotic cardiovascular disease events for individuals of White/European and non-White/European ethnicity using cardiovascular risk calculators. **eTable 6. **Additional value of lipoprotein(a) metrics to atherosclerotic cardiovascular disease risk discrimination among individuals of White/European ethnicity defined as borderline-to-intermediate risk by QRISK3 using only complete data. **eTable 7. **Additional value of lipoprotein(a) metrics to atherosclerotic cardiovascular disease risk discrimination among individuals of White/European ethnicity defined by the Pooled Cohort Equations as borderline-intermediate risk using complete and imputed data. **eTable 8. **Additional value of lipoprotein(a) metrics to atherosclerotic cardiovascular disease risk discrimination among individuals of White/European ethnicity defined by the Pooled Cohort Equations as borderline-intermediate risk using complete data. **eFigure 1. **Self-reported ethnicity subgroups cluster with genetic principal components of ancestry. **eFigure 2. **Distributions of measured lipoprotein(a) and LPA genetic risk scores. **eFigure 3. **Association of measured lipoprotein(a) and LPA genetic risk score with risk of incident, composite atherosclerotic cardiovascular events. **eFigure 4. **Measured lipoprotein(a) and LPA genetic risk score (GRS) associate with incident atherosclerotic cardiovascular disease among individuals of White/European ethnicity using cholesterol lowering medication. **eFigure 5. **Measured lipoprotein(a) has stronger associations with composite atherosclerotic cardiovascular disease events at the extremes of elevated lipoprotein(a) than the LPA genetic score. **eFigure 6. **Distributions of atherosclerotic cardiovascular disease risk determined by clinical risk calculators. **eReferences. **
