# Supplementary material for: Clinical Utility of Lipoprotein(a) and LPA Genetic Risk Score in Risk Prediction of Incident Atherosclerotic Cardiovascular Disease
Source: JAMA Cardiol. 2020 Oct 6;6(3):1–9. doi: 10.1001/jamacardio.2020.5398 (PMC7539232; doi:10.1001/jamacardio.2020.5398)
Supplement: Supplement. — eMethods. eTable 1. Genetic variants used in the LPA genetic risk score. eTable 2. International Statistical Classification of Diseases and Related Health Problems (ICD) and Office of Population Censuses and Surveys Classification of Interventions and Procedures (OCPS) codes used to define atherosclerotic cardiovascular disease events. eTable 3. Spearman's rank correlation between LPA genetic risk score and measured lipoprotein(a) levels for different ethnic groups. eTable 4. Enrollment characteristics of UK Biobank study participants not using cholesterol-lowering medication and without prevalent atherosclerotic cardiovascular disease at study enrollment. eTable 5. Risk discrimination models for incident atherosclerotic cardiovascular disease events for individuals of White/European and non-White/European ethnicity using cardiovascular risk calculators. eTable 6. Additional value of lipoprotein(a) metrics to atherosclerotic cardiovascular disease risk discrimination among individuals of White/European ethnicity defined as borderline-to-intermediate risk by QRISK3 using only complete data. eTable 7. Additional value of lipoprotein(a) metrics to atherosclerotic cardiovascular disease risk discrimination among individuals of White/European ethnicity defined by the Pooled Cohort Equations as borderline-intermediate risk using complete and imputed data. eTable 8. Additional value of lipoprotein(a) metrics to atherosclerotic cardiovascular disease risk discrimination among individuals of White/European ethnicity defined by the Pooled Cohort Equations as borderline-intermediate risk using complete data. eFigure 1. Self-reported ethnicity subgroups cluster with genetic principal components of ancestry. eFigure 2. Distributions of measured lipoprotein(a) and LPA genetic risk scores. eFigure 3. Association of measured lipoprotein(a) and LPA genetic risk score with risk of incident, composite atherosclerotic cardiovascular events. eFigure 4. Measured lipoprotein(a) and LPA ge [file jamacardiol-e205398-s001.pdf]

Supplemental Online Content

Trinder M, Uddin MM, Finneran P, Aragam KG, Natarajan P. Clinical utility of lipoprotein(a) and *LPA* genetic risk score in risk prediction of incident atherosclerotic cardiovascular disease. *JAMA Cardiol*. Published online October 6, 2020. doi:10.1001/jamacardio.2020.5398

eMethods.
eTable 1. Genetic variants used in the LPA genetic risk score.
eTable 2. International Statistical Classification of Diseases and Related Health Problems (ICD) and Office of Population Censuses and Surveys Classification of Interventions and Procedures (OCPS) codes used to define atherosclerotic cardiovascular disease events.
eTable 3. Spearman's rank correlation between LPA genetic risk score and measured lipoprotein(a) levels for different ethnic groups.
eTable 4. Enrollment characteristics of UK Biobank study participants not using cholesterol-lowering medication and without prevalent atherosclerotic cardiovascular disease at study enrollment.
eTable 5. Risk discrimination models for incident atherosclerotic cardiovascular disease events for individuals of White/European and non-White/European ethnicity using cardiovascular risk calculators.
eTable 6. Additional value of lipoprotein(a) metrics to atherosclerotic cardiovascular disease risk discrimination among individuals of White/European ethnicity defined as borderline-to-intermediate risk by QRISK3 using only complete data.
eTable 7. Additional value of lipoprotein(a) metrics to atherosclerotic cardiovascular disease risk discrimination among individuals of White/European ethnicity defined by the Pooled Cohort Equations as borderline-intermediate risk using complete and imputed data.
eTable 8. Additional value of lipoprotein(a) metrics to atherosclerotic cardiovascular disease risk discrimination among individuals of White/European ethnicity defined by the Pooled Cohort Equations as borderline-intermediate risk using complete data.
eFigure 1. Self-reported ethnicity subgroups cluster with genetic principal components of ancestry.
eFigure 2. Distributions of measured lipoprotein(a) and LPA genetic risk scores.
eFigure 3. Association of measured lipoprotein(a) and LPA genetic risk score with risk of incident, composite atherosclerotic cardiovascular events.
eFigure 4. Measured lipoprotein(a) and LPA genetic risk score (GRS) associate with incident atherosclerotic cardiovascular disease among individuals of White/European ethnicity using cholesterol lowering medication.
eFigure 5. Measured lipoprotein(a) has stronger associations with composite atherosclerotic cardiovascular disease events at the extremes of elevated lipoprotein(a) than the LPA genetic score.
eFigure 6. Distributions of atherosclerotic cardiovascular disease risk determined by clinical risk calculators.
eReferences.

This supplemental material has been provided by the authors to give readers additional information about their work.

© 2020 Trinder M et al. *JAMA Cardiology.*

ONLINE-ONLY METHODS

LPA genetic risk score.

LPA genetic risk scores were calculated using the external weights for 43 single-nucleotide variants (SNVs) described by Burgess et al. (2018) (Online-Only Table 1)[1]. Quality control filtering of genotyping individuals from the UK Biobank was performed such that individuals that were a mismatch between genetic and reported sex, outliers for heterozygosity or missing rate, or missing more than 3 SNVs from the *LPA* genetic risk score were excluded. *LPA* genetic risk scores were calculated using the weighted formula Σ [βx * SNVx], where βx is the effect size for the lipprotein(a)-associated effect and SNV_x is the number of effect alleles at that locus for SNVx (0, 1, or 2).

© 2020 Trinder M et al. *JAMA Cardiology.*

ONLINE-ONLY TABLES

Online-Only Table 1. Genetic variants used in the *LPA* genetic risk score. External weights for the association between genetic variants and lipoprotein(a) were obtained from Burgess et al. (2018)[1]. Values for the association with lipoprotein(a) were converted from mg/dL to nmol/L by multiplying by 2.15.

Chr:Pos (hg19)	rsID	Effect allele	Other allele	Conditional association with Lp(a), Beta (nmol/L)	Conditional association with Lp(a), SE (nmol/L)
6:160997118	rs74617384	T	A	91.16	1.075
6:161013013	rs140570886	C	T	172.43	1.72
6:161017363	rs73596816	A	G	41.28	1.29
6:160891897	rs182443492	A	C	79.12	2.15
6:161032800	rs369686024	A	G	41.28	1.72
6:161089307	rs56393506	T	C	26.66	0.86
6:160831796	rs151135411	A	G	149.425	5.805
6:161292838	rs145099029	C	A	38.27	3.87
6:160998199	rs41267813	A	G	-126.42	6.235
6:160890350	rs6916433	T	A	-10.105	0.645
6:161137990	rs783147	A	G	-4.3	0.645
6:160953137	rs41266379	C	T	15.265	1.505
6:160954800	rs143461353	T	C	28.165	2.15
6:160942926	rs142126734	A	G	16.125	1.075
6:160899049	rs139609547	-	A	9.46	0.86
6:161162290	rs1835346	G	A	11.18	1.505
6:161159366	rs4252152	G	T	19.565	1.935
6:161078894	rs79246098	C	T	13.33	1.935
6:160966559	rs139145675	A	G	-48.375	5.16
6:161022107	rs41259144	T	C	-20.64	1.72
6:161012805	rs9456551	C	T	7.74	0.43
6:160953642	rs41267809	G	A	-14.19	1.29
6:161257953	rs34371670	T	C	-18.06	1.505
6:161070653	rs41269876	A	C	-17.63	1.29
6:160909667	rs141834709	A	T	18.705	2.15
6:161162406	rs4252170	C	T	6.88	0.86
6:161251940	rs138491411	G	A	10.75	1.72
6:160720804	rs183815886	C	G	31.82	3.87
6:160847571	rs117446263	A	G	-11.18	1.29
6:160543317	rs200684404	T	C	145.555	19.78
6:160493099	rs200144324	T	C	175.225	24.295

© 2020 Trinder M et al. *JAMA Cardiology.*

6:161087652	rs77337569	G	T	11.18	1.72
6:161214526	rs186418835	A	G	-20.855	3.225
6:161177443	rs117534432	T	C	7.095	1.075
6:161011999	rs200376184	C	G	37.625	5.805
6:161189071	rs11753588	A	G	-5.16	0.645
6:161285760	rs4709474	G	A	3.655	0.43
6:161031132	rs191690882	A	G	-28.38	4.085
6:161255668	rs182349273	G	A	73.96	12.255
6:161088956	rs75274517	A	G	-13.975	2.15
6:160825930	rs143365644	T	A	7.955	1.075
6:161135746	rs139389770	G	T	-11.18	1.935
6:161250301	rs140606700	G	A	13.76	2.58

© 2020 Trinder M et al. *JAMA Cardiology.*

Online-Only Table 1. International Statistical Classification of Diseases and Related Health Problems (ICD) and Office of Population Censuses and Surveys Classification of Interventions and Procedures (OCPS) codes used to define atherosclerotic cardiovascular disease events.

**Cardiovascular Outcome Category**	**ICD-9 Diagnosis Codes**	**ICD-10 Diagnosis Codes**	**OCPS-4 (operations)**
Myocardial infarction	• 410 Acute myocardial infarction<br>• 411 Other acute and subacute forms of ischaemic heart disease<br>• 412 Old myocardial infarction<br>• 42979 Ill-defined descriptions and complications of heart disease - Other	• I21 Acute myocardial infarction<br>• I22 Subsequent myocardial infarction<br>• I23 Certain current complications following acute myocardial infarction<br>• I24.1 Dressler syndrome<br>• I25.2 Old myocardial infarction.	N/A
Ischemic stroke	• 434 Occlusion of cerebral arteries<br>• 436 Acute, but ill-defined, cerebrovascular disease	• I63 Cerebral infarction<br>• I64 Stroke, not specified as haemorrhage or infarction.	N/A
Cardiovascular mortality	N/A	Any diagnosis code for causes of death related to: *Chapter IX Diseases of the circulatory system*	N/A
Coronary artery disease	Myocardial infarction codes	Myocardial infarction codes	• K40 Saphenous vein graft replacement of coronary artery<br>• K41 Other autograft replacement of coronary artery<br>• K42 Allograft replacement of coronary artery<br>• K43 Prosthetic replacement of coronary artery<br>• K44 Other replacement of coronary artery<br>• K45 Connection of thoracic artery to coronary artery<br>• K46 Other bypass of coronary artery<br>• K47 Repair of coronary artery<br>• K48 Other open operations on coronary artery<br>• K49 Transluminal balloon angioplasty of coronary artery

© 2020 Trinder M et al. *JAMA Cardiology.*

			• K50 Other therapeutic transluminal operations on coronary artery<br>• K75 Percutaneous transluminal balloon angioplasty and insertion of stent into coronary artery
Peripheral artery disease (non-coronary or cerebrovascular events)	• 440 Atherosclerosis<br>• 444 Arterial embolism and thrombosis<br>• 4438 Other specified peripheral vascular disease<br>• 4439 Peripheral vascular disease, unspecified	• I70 Atherosclerosis<br>• I74 Arterial embolism and thrombosis<br>• I73.8 Other specified peripheral vascular diseases<br>• I73.9 Peripheral vascular disease, unspecified	• L50 Other emergency bypass of iliac artery<br>• L51 Other bypass of iliac artery<br>• L52 Reconstruction of iliac artery<br>• L54 Transluminal operations on iliac artery<br>• L58 Other emergency bypass of femoral artery<br>• L59 Other bypass of femoral artery<br>• L60 Reconstruction of femoral artery<br>• L63 Transluminal operations on femoral artery<br>• X09 Amputation of leg

© 2020 Trinder M et al. *JAMA Cardiology.*

Online-Only Table 3. Spearman's rank correlation between *LPA* genetic risk score and measured lipoprotein(a) levels for different ethnic groups. Black/African (AFR: Caribbean, African, any other Black background), White/European (EUR: British, Irish, any other White background), East Asian (EAS: Chinese, other Asian background), South Asian (SAS: Indian, Pakistani, Bangladeshi), unknown (other ethnic group, do not know, or prefer not to answer).

**Ethnic Group**	**n**	**Correlation Co-efficient**	**p-value**
Admixed	2,340	0.483	$< 2.2 \times 10^{-16}$
Black/African (AFR)	6,521	0.070	1.76×10^{-8}
East Asian (EAS)	2,774	0.281	$< 2.2 \times 10^{-16}$
White/European (EUR)	350,903	0.717	$< 2.2 \times 10^{-16}$
South Asian (SAS)	6,203	0.371	$< 2.2 \times 10^{-16}$
Unknown	5,358	0.437	$< 2.2 \times 10^{-16}$

Online-Only Table 4. Enrollment characteristics of UK Biobank study participants not using cholesterol-lowering medication and without prevalent atherosclerotic cardiovascular disease at study enrollment. High-density lipoprotein cholesterol (HDL-C), low-density lipoprotein cholesterol (LDL-C).

**Characteristic**	**Measure**	**White/European and Non-White/European**	**European**
n	no.	300839	283540
Age (years)	mean (SD)	56.6 (8.0)	56.8 (8.0)
Female sex	no. (%)	174555 (58.0)	164734 (58.1)
Ethnicity - Admixed	no. (%)	1991 (0.7)	0 (0.0)
Ethnicity - African/Black	no. (%)	5163 (1.7)	0 (0.0)
Ethnicity - East Asian	no. (%)	2105 (0.7)	0 (0.0)
Ethnicity - European	no. (%)	283540 (94.2)	283540 (100.0)
Ethnicity - South Asian	no. (%)	4275 (1.4)	0 (0.0)
Ethnicity - Unkown	no. (%)	3765 (1.3)	0 (0.0)
Total cholesterol (mg/dL)	mean (SD) / n	229.3 (41.2) / 300650	230.1 (41.1) / 283360
Direct LDL-C (mg/dL)	mean (SD) / n	144.5 (31.5) / 300167	145.0 (31.4) / 282907
Triglycerides (mg/dL)	median (IQR) / n	127.0 (93.4) / 300636	127.6 (93.4) / 283346
HDL-C (mg/dL)	mean (SD) / n	57.4 (14.7) / 275219	57.6 (14.7) / 259349
Lipoprotein(a) (nmol/L)	median (IQR) / n	24.1 (73.6)	23.1 (72.9)
Lipoprotein(a) ≥ 120 nmol/L	no. (%)	53960 (17.9)	50961 (18.0)
C-reactive protein (g/L)	median (IQR) / n	1.3 (2.1) / 300037	1.3 (2.0) / 282779
Hemoglobin A1c (mmol/mol)	median (IQR) / n	34.9 (4.7) / 300037	34.7 (4.7) / 270823
Antihypertensive medication	no. (%) / n	36255 (12.1) / 300839	33493 (11.8) / 283540
Hypertension	no. (%) / n	59447 (19.8) / 300344	55562 (19.6) / 283140
Diabetes mellitus	no. (%) / n	4318 (1.4) / 300237	3525 (1.2) / 283088
Body mass index (kg/m^2)	mean (SD) / n	27.0 (4.6) / 299831	26.9 (4.6) / 282759
Current smoker	no. (%) / n	30628 (10.2) / 299851	28527 (10.1) / 282669

Online-Only Table 5. Risk discrimination models for incident atherosclerotic cardiovascular disease events for individuals of European and non-European ethnicity using cardiovascular risk calculators. The risk prediction of QRISK3 and the Pooled Cohort Equations were compared for the overall study cohort of 300,839 individuals of European and non-European ethnicities. 11,731 individuals experienced a myocardial infarction, an ischemic stroke, or cardiovascular mortality event over a median follow-up of 11.1 years (interquartile range 1.4 years). The area under the receiver operating characteristic curve (AUROC) and the Harrell's C-statistic for Cox proportional-hazards models are shown for QRISK3 and the PCE. Models were compared using A DeLong's test for receiver operating characteristic curves and an analysis of variance test for Cox proportional-hazard models.

**Features**	**AUROC (95% CI) [p-value]**	**Harrell's C-statistic (SE) [p-value]**
Pooled Cohort Equations	0.742 (0.738-0.747) [reference]	0.741 (0.002) [reference]
QRISK3	0.749 (0.745-0.754) [3.31×10^{-15}]	0.748 (0.002) [$p<2.2 \times 10^{-16}$]

Online-Only Table 6. Additional value of lipoprotein(a) metrics to atherosclerotic cardiovascular disease risk discrimination among individuals of White/European ethnicity defined as borderline-to-intermediate risk by QRISK3 using only complete data. This subgroup included 121,428 individuals classified as having borderline-intermediate ASCVD risk without prevalent ASCVD, diabetes mellitus, severe hypercholesterolemia, or use of cholesterol-lowering medication (10-year risk of 5-20%). 4,585 individuals experienced a myocardial infarction, an ischemic stroke, or cardiovascular mortality event over a median follow-up of 11.0 years (interquartile range 1.4 years). The area under the receiver operating characteristic curve (AUROC) and the Harrell's C-statistic for Cox proportional-hazards models are shown for the Pooled Cohort Equations (PCE) with and without the addition of continuous measured lipoprotein(a) and *LPA* genetic risk score (GRS). Models were compared relative to the QRISK3 model using an analysis of variance test for Cox proportional-hazard models or DeLong's test for receiver operating characteristic curves. Standard error (SE).

**Features**	**AUROC (SE) [p-value]**	**Harrell's C-statistic (SE) [p-value]**
QRISK3	0.640 (0.632-0.648) [ref.]	0.639 (0.004) [ref.]
QRISK3 & Lp(a)	0.642 (0.634-0.650) [p=0.02]	0.641 (0.004) [p=3.54×10^{-8}]
QRISK3 & *LPA* GRS	0.642 (0.634-0.650) [p=0.02]	0.641 (0.004) [p=1.20×10^{-8}]
QRISK3 & Lp(a) & *LPA* GRS	0.642 (0.634-0.650) [p=0.01]	0.641 (0.004) [p=1.95×10^{-8}]

Online-Only Table 7. Additional value of lipoprotein(a) metrics to atherosclerotic cardiovascular disease risk discrimination among individuals of White/European ethnicity defined by the Pooled Cohort Equations as borderline-intermediate risk using complete and imputed data. This subgroup included 113,703 individuals classified as having borderline-intermediate ASCVD risk without prevalent ASCVD, diabetes mellitus, severe hypercholesterolemia, or use of cholesterol-lowering medication (10-year risk of 5-20%). 5,938 individuals experienced a myocardial infarction, an ischemic stroke, or cardiovascular mortality event over a median follow-up of 11.1 years (interquartile range 1.5 years). The area under the receiver operating characteristic curve (AUROC) and the Harrell's C-statistic for Cox proportional-hazards models are shown for the Pooled Cohort Equations (PCE) with and without the addition of continuous measured lipoprotein(a) and *LPA* genetic risk score (GRS). Models were compared relative to the PCE model using an analysis of variance test for Cox proportional-hazard models or DeLong's test for receiver operating characteristic curves. Standard error (SE).

**Features**	**AUROC (SE)<br>[p-value]**	**Harrell's C-statistic (SE)<br>[p-value]**
PCE	0.608 (0.601-0.615) [ref.]	0.609 (0.004) [ref.]
PCE & Lp(a)	0.611 (0.603-0.618) [p=0.01]	0.611 (0.004) [$3.71x10^{-14}$]
PCE & *LPA* GRS	0.611 (0.603-0.617) [p=0.03]	0.611 (0.004) [$5.48x10^{-10}$]
PCE & Lp(a) & *LPA* GRS	0.611 (0.603-0.617) [p=0.01]	0.611 (0.004) [$3.03x10^{-13}$]

Online-Only Table 8. Additional value of lipoprotein(a) metrics to atherosclerotic cardiovascular disease risk discrimination among individuals of White/European ethnicity defined by the Pooled Cohort Equations as borderline-intermediate risk using complete data. This subgroup included 96,260 individuals classified as having borderline-intermediate ASCVD risk without prevalent ASCVD, diabetes mellitus, severe hypercholesterolemia, or use of cholesterol-lowering medication (10-year risk of 5-20%). 5,436 individuals experienced a myocardial infarction, an ischemic stroke, or cardiovascular mortality over a median follow-up of 11.0 years (interquartile range 1.5 years). The area under the receiver operating characteristic curve (AUROC) and the Harrell's C-statistic for Cox proportional-hazards models are shown for the Pooled Cohort Equations (PCE) with and without the addition of continuous measured lipoprotein(a) and *LPA* genetic risk score (GRS). Models were compared relative to the PCE model using an analysis of variance test for Cox proportional-hazard models or DeLong's test for receiver operating characteristic curves. Standard error (SE).

**Features**	**AUROC (SE) [p-value]**	**Harrell's C-statistic (SE) [p-value]**
PCE	0.614 (0.609-0.624) [reference]	0.617 (0.004) [reference]
PCE & Lp(a)	0.619 (0.611-0.627) [p=0.03]	0.619 (0.004) [p=$1.44x10^{-11}$]
PCE & *LPA* GRS	0.619 (0.611-0.626) [p=0.02]	0.619 (0.004) [p=$2.20x10^{-8}$]
PCE & Lp(a) & *LPA* GRS	0.619 (0.611-0.627) [p=0.02]	0.619 (0.004) [p=$1.03x10^{-10}$]

ONLINE-ONLY FIGURES

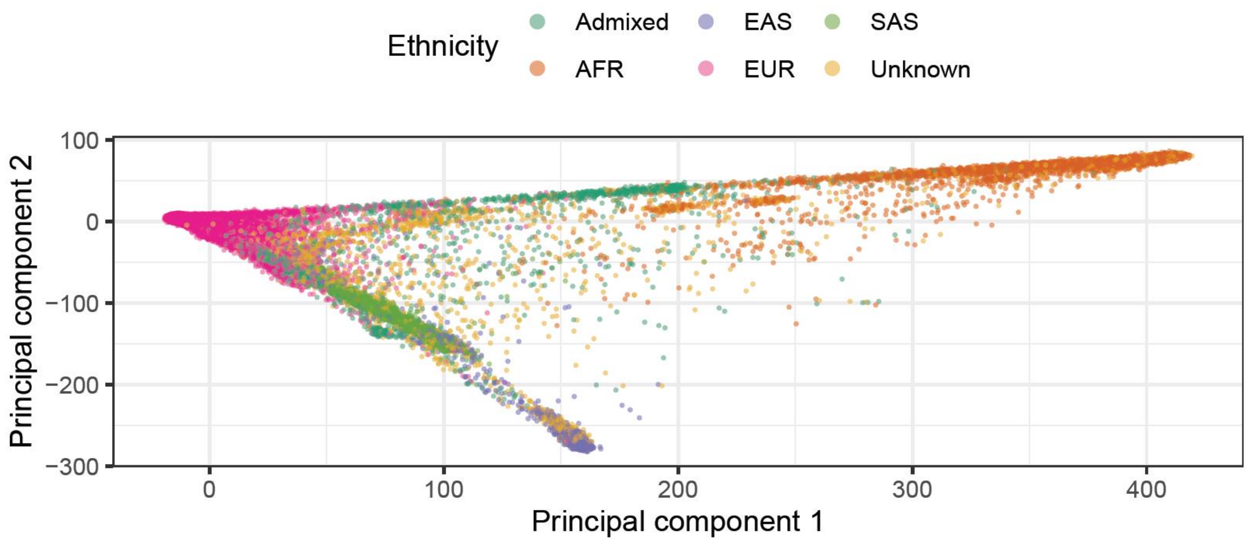

Online-Only Figure 1. Self-reported ethnicity subgroups cluster with genetic principal components of ancestry. Individuals of admixed, Black/African (AFR: Caribbean, African, any other Black background), White/European (EUR: British, Irish, any other White background), East Asian (EAS: Chinese, other Asian background), South Asian (SAS: Indian, Pakistani, Bangladeshi), and unknown (other ethnic group, do not know, or prefer not to answer) self-reported ethnicity are depicted based on the first 2 principal components of genetic ancestry.

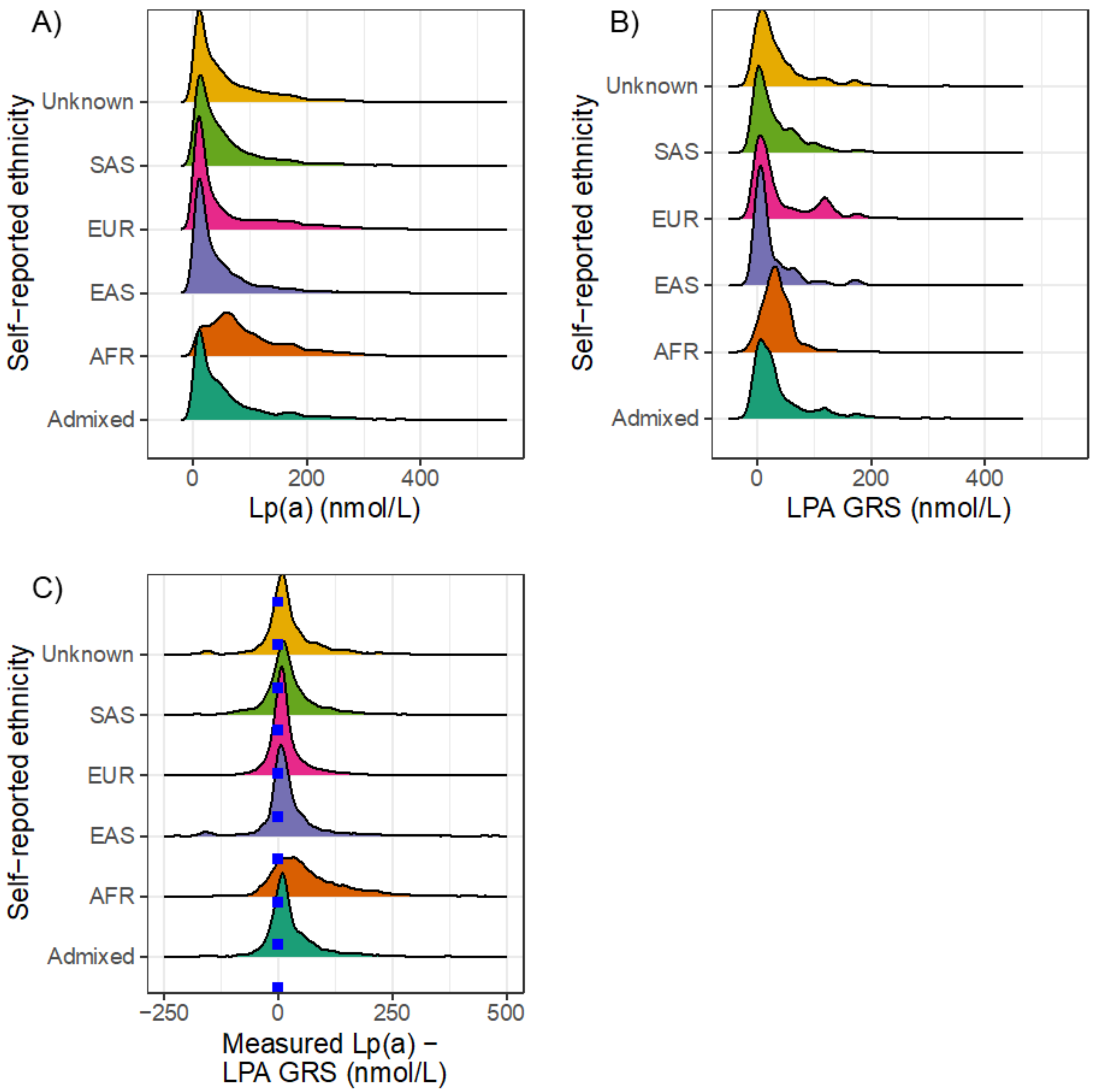

Online-Only Figure 2. Distributions of measured lipoprotein(a) and *LPA* genetic risk scores. The distributions of (A) measured lipoprotein(a) [Lp(a)], (B) lipoprotein(a) genetic risk score (GRS), and (C) residuals of measured lipoprotein(a) – *LPA* genetic risk score are depicted for ethnic groupings. Black/African (AFR: Caribbean, African, any other Black background), White/European (EUR: British, Irish, any other White background), East Asian (EAS: Chinese, other Asian background), South Asian (SAS: Indian, Pakistani, Bangladeshi), unknown (other ethnic group, do not know, or prefer not to answer).

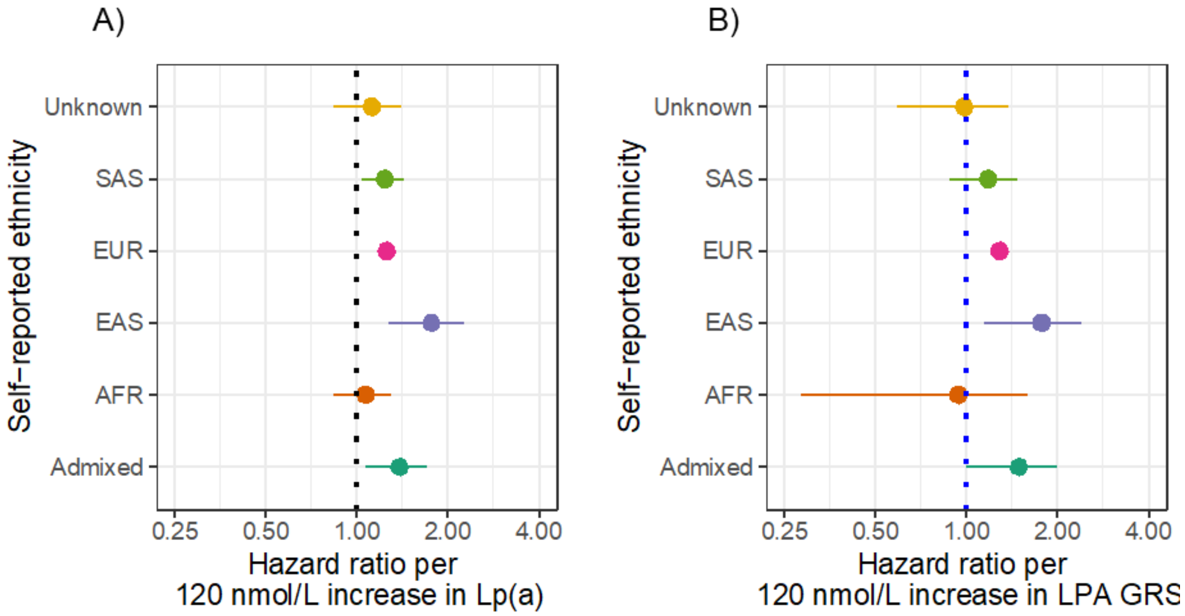

Online-Only Figure 3. Association of measured lipoprotein(a) and *LPA* genetic risk score with risk of incident, composite atherosclerotic cardiovascular events. The risk of incident atherosclerotic cardiovascular disease (ASCVD) events, stratified by self-reported ethnicity is depicted as hazard ratios and 95% confidence intervals for the composite endpoints of peripheral arterial disease, ischemic stroke, coronary artery disease, myocardial infarction, and cardiovascular disease mortality for individuals not using cholesterol-lowering medication and without prevalent ASCVD at enrollment. Hazard ratios are scaled to depict a 120 nmol/L increase in (A) measured lipoprotein(a) (Lp(a)) or (B) *LPA* genetic risk score (GRS) levels. Cox proportional hazard models included age, sex, assessment centre, genotyping batch, and the first five principal components of ancestry. Black/African (AFR: Caribbean, African, any other Black background), White/European (EUR: British, Irish, any other White background), East Asian (EAS: Chinese, other Asian background), South Asian (SAS: Indian, Pakistani, Bangladeshi), unknown (other ethnic group, do not know, or prefer not to answer).

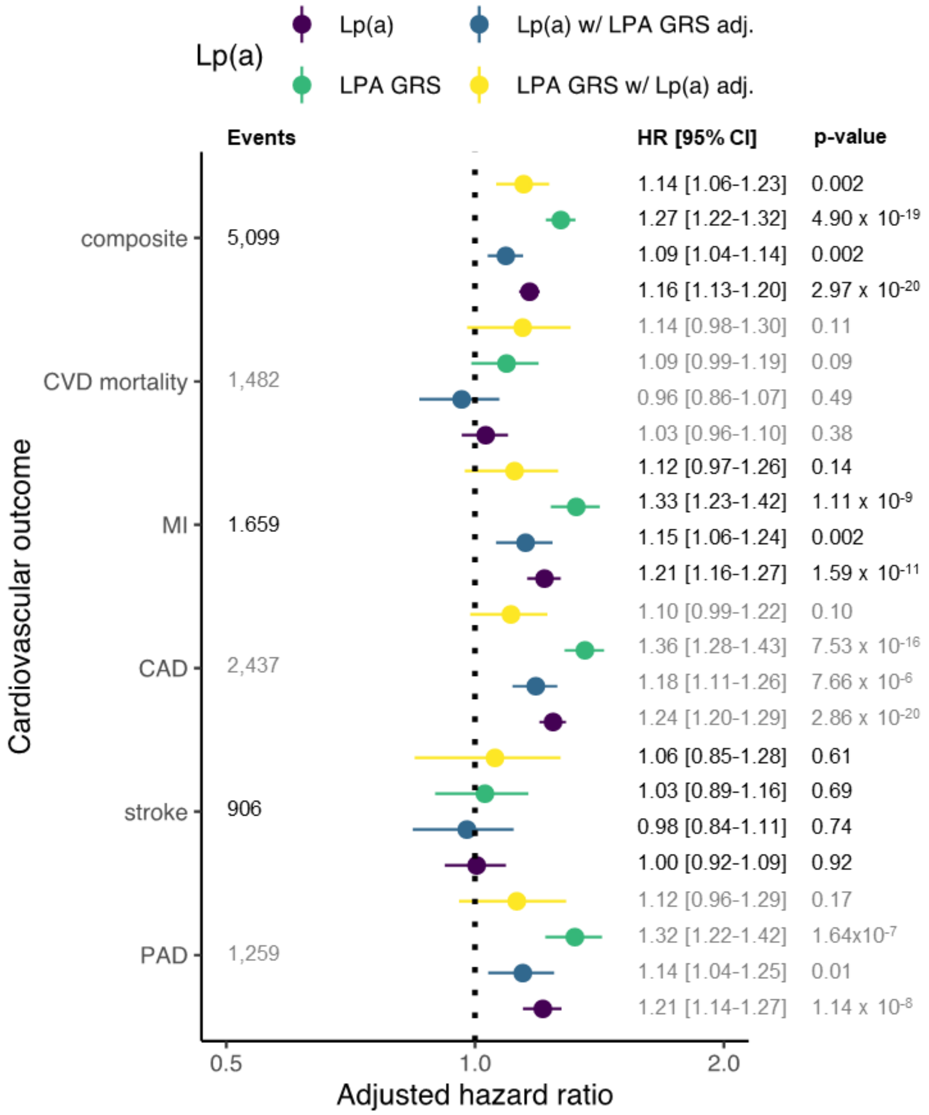

Online-Only Figure 4. Measured lipoprotein(a) and *LPA* genetic risk score (GRS) associate with incident atherosclerotic cardiovascular disease among individuals of White/European ethnicity using cholesterol lowering medication. The risk of incident atherosclerotic cardiovascular disease (ASCVD) events is depicted as hazard ratios and 95% confidence intervals for peripheral arterial disease, ischemic stroke, coronary artery disease, myocardial infarction, cardiovascular disease (CVD) mortality, and composite ASCVD for 43,829 individuals without prevalent ASCVD, but using cholesterol-lowering medication at enrollment. Hazard ratios are scaled to depict a 120 nmol/L increase in measured lipoprotein(a) (Lp(a)) or *LPA* genetic risk score (GRS) levels. All Cox proportional hazard models included age, sex, assessment centre, genotyping batch, and the first five principal components of ancestry.

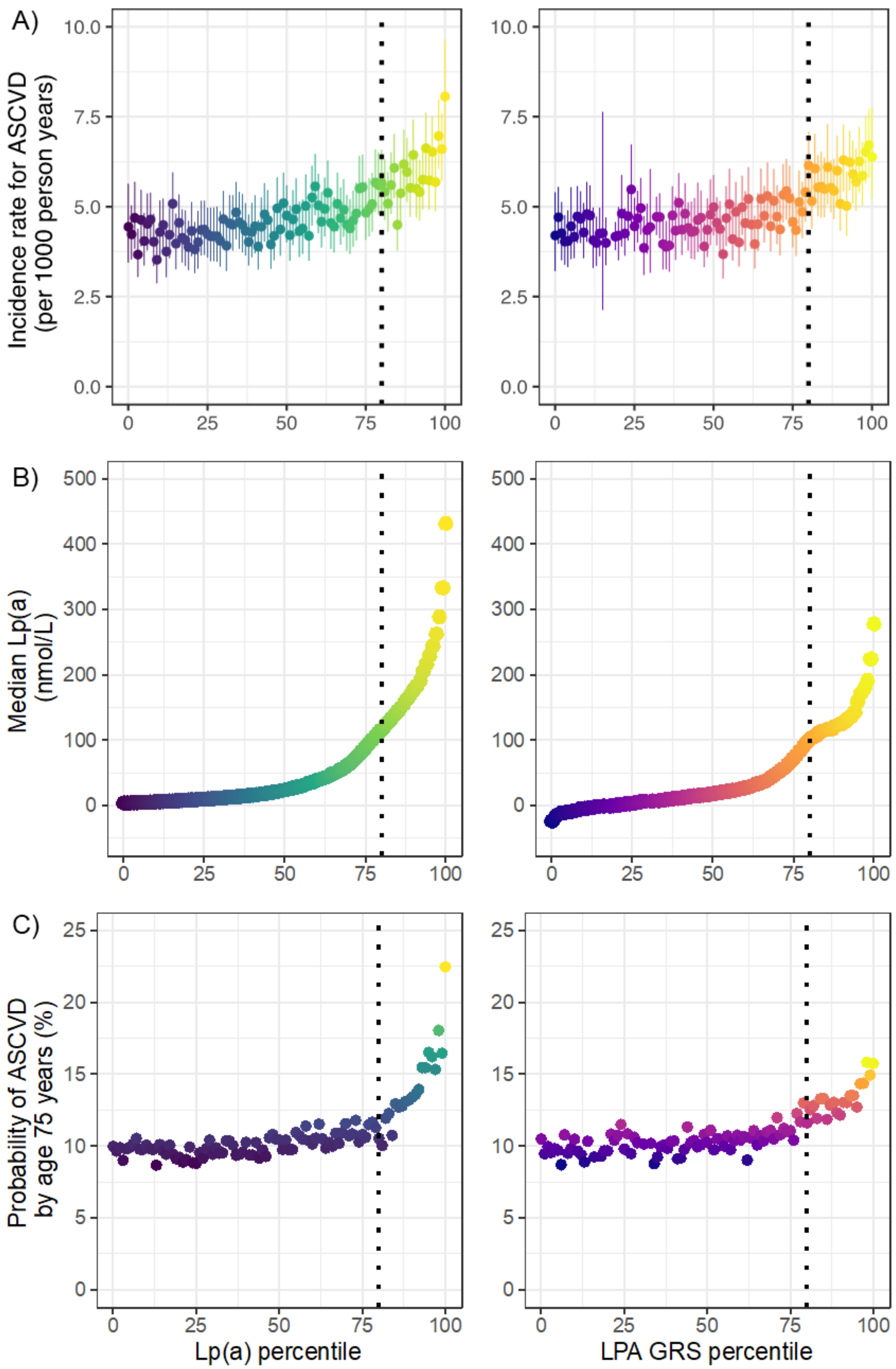

A)
B)
C)
Incidence rate for ASCVD (per 1000 person years)
Median Lp(a) (nmol/L)
Probability of ASCVD by age 75 years (%)
Lp(a) percentile
LPA GRS percentile

Online-Only Figure 5. Measured lipoprotein(a) has stronger associations with composite atherosclerotic cardiovascular disease events at the extremes of elevated lipoprotein(a) than the *LPA* genetic score. (A) The incidence rate of composite atherosclerotic cardiovascular disease (ASCVD) per 1000 person years (± 95% CIs), (B) measured levels of lipoprotein(a) (± interquartile range), and (C) probability of composite ASCVD by 75 years-of-age are displayed for each percentile of measured lipoprotein(a) and *LPA* genetic risk score (GRS). Dashed lines correspond to the 80th percentile of measured lipoprotein and *LPA* GRS (approximately 120 nmol/L). Associations with incidence rates were determined from 283,540 individuals of White/European ethnicity not using cholesterol-lowering medication and without prevalent ASCVD at enrollment. Alternatively, associations with measured lipoprotein(a) and probability of ASCVD were determined from 350,903 individuals of White/European ethnicity regardless of cholesterol-lowering medication use or prevalent ASCVD at enrollment.

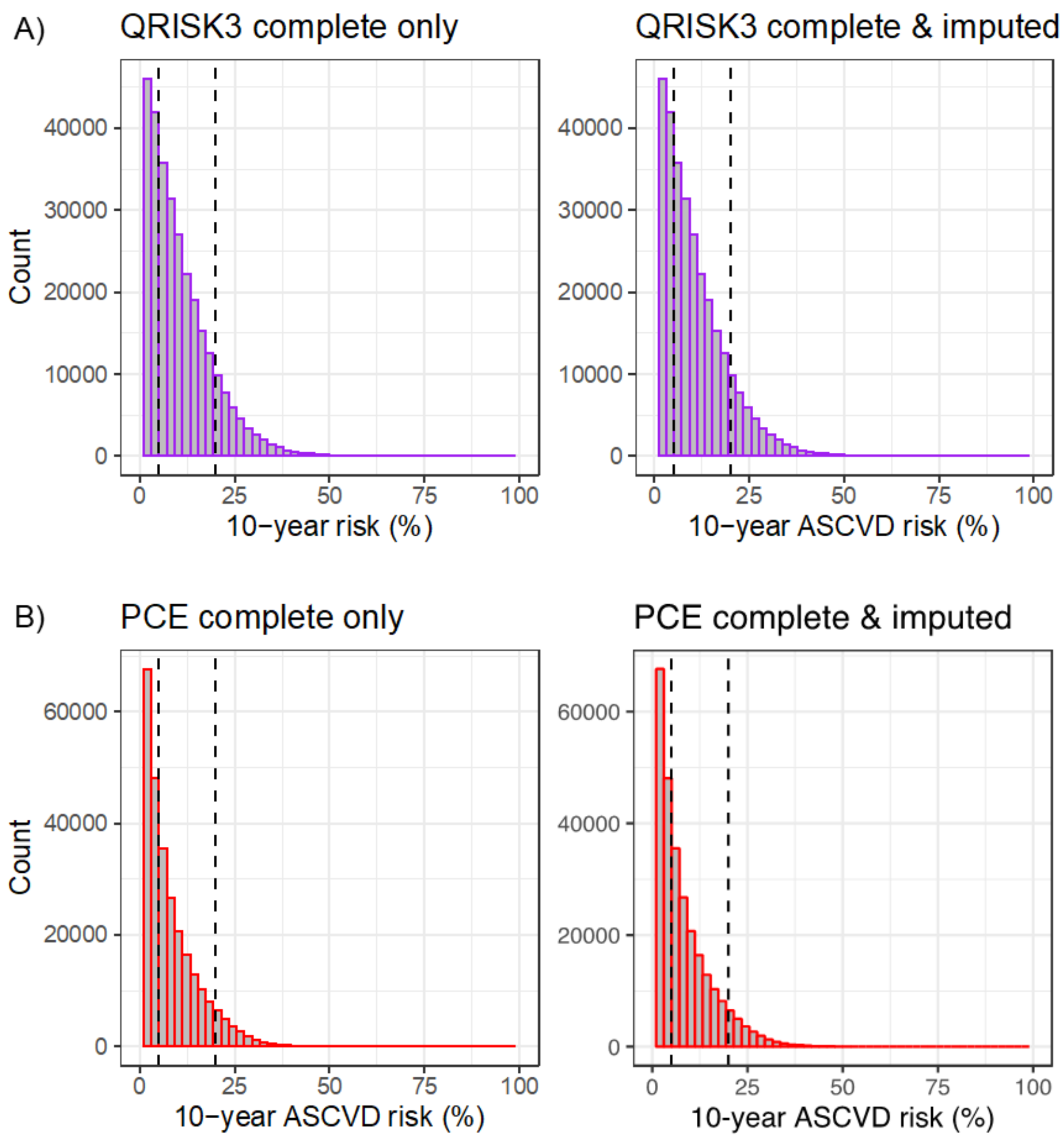

Online-Only Figure 6. Distributions of atherosclerotic cardiovascular disease risk determined by clinical risk calculators. The distribution of 10-year atherosclerotic cardiovascular disease (ASCVD) risk determined by (A) QRISK3 and (B) the Pooled Cohort Equations is displayed for 300,839 individuals of White/European and non-White/European individuals not using cholesterol-lowering medication and without prevalent ASCVD at enrollment. Data are displayed using complete data only (QRISK3: 254,315; PCE: n=255,510) and complete and imputed data (QRISK3 and QRISK3: n=300,839). The dashed black lines depict 5% (low-to-borderline) and 20% (intermediate-to-high) ASCVD risk thresholds.

ONLINE-ONLY REFERENCES

1. Burgess S, Ference BA, Staley JR, Freitag DF, Mason AM, Nielsen SF, et al. Association of *LPA* variants with risk of coronary disease and the implications for lipoprotein(a)-lowering therapies: a Mendelian randomization analysis. *JAMA Cardiol*. 2018;3(7):619-627.
